# A Comprehensive Methodology for Microbial Strain Typing Using Fourier-Transform Infrared Spectroscopy

**DOI:** 10.3390/mps7030048

**Published:** 2024-06-11

**Authors:** Francis Muchaamba, Roger Stephan

**Affiliations:** Institute for Food Safety and Hygiene, Vetsuisse Faculty, University of Zurich, Winterthurerstrasse 272, CH-8057 Zurich, Switzerland

**Keywords:** strain typing, Fourier-transform infrared spectroscopy, outbreak, surveillance, pathogen detection

## Abstract

Timely and accurate detection and characterization of microbial threats is crucial for effective infection and outbreak management. Additionally, in food production, rapid microbe identification is indispensable for maintaining quality control and hygiene standards. Current methods for typing microbial strains often rely on labor-intensive, time-consuming, and expensive DNA- and sera-serotyping techniques, limiting their applicability in rapid-response scenarios. In this context, the IR Biotyper^®^, utilizing Fourier-transform infrared (FTIR) spectroscopy, offers a novel approach, providing specific spectra for fast strain typing within 3 h. This methodology article serves as a comprehensive resource for researchers and technicians aiming to utilize FTIR spectroscopy for microbial strain typing. It encompasses detailed guidelines on sample preparation, data acquisition, and analysis techniques, ensuring the generation of reliable and reproducible results. We highlight the IR Biotyper^®^’s rapid and accurate discrimination capabilities, showcasing its potential for real-time pathogen monitoring and source-tracking to enhance public health and food safety. We propose its integration as an early screening method, followed by more detailed analysis with whole-genome sequencing, to optimize detection accuracy and response efficiency in microbial surveillance systems.

## 1. Introduction

Increased food production industrialization, international trade, global warming, and changes to eating habits towards ready-to-eat foods have created a great challenge to ensure food safety [1,2]. This is coupled with increased virulence and tolerance to hurdles by foodborne pathogens, which has necessitated an increased demand for rapid food analysis to ensure the safety and quality of food [3,4]. Food contamination with pathogens such as *Listeria monocytogenes*, *Cronobacter sakazakii*, Shiga-toxin-producing *Escherichia coli*, and *Salmonella* spp. is a major public health threat [5,6,7]. Rapid and early detection of these microbial contaminants allows the timely implementation of appropriate measures to terminate outbreaks and prevent further transmission and morbidity [8]. This requires fast, affordable, and reliable tools for identification and classification of such threats. The emergence of new microbial threats, in part due to adaptation of microorganisms, climate change, modifications of human lifestyle and demographics, changes in economic development, excessive land use, and increasing environmental pollution, has exacerbated this need [9,10].

Understanding the diversity and characteristics of microbial strains is of paramount importance in various fields, ranging from public health to industrial manufacturing. Strain identification plays a critical role in outbreak investigation, epidemiology, food safety, factory hygiene programs, vaccine development, disease detection, and treatment modality decisions. Each microbial strain possesses unique genetic and phenotypic traits that influence its pathogenicity, antibiotic resistance profile, and environmental adaptability. Therefore, accurate and rapid strain typing methodologies are indispensable for effective management and control of microbial infections and other related challenges.

Several tools are being applied for pathogen detection to prevent and control outbreaks. Pulsed-field gel electrophoresis (PFGE) and multi-locus sequence typing (MLST) are well-established typing methods in microbiology laboratories [8,11,12], but have limitations. PFGE is generally costly, labor-intensive, and time-consuming. MLST has excellent reproducibility but is expensive and limited to applicable strains [11]. Matrix-assisted laser desorption/ionization-time of flight (MALDI-TOF) mass spectrometry (MS) has revolutionized and reduced the time required for pathogen identification [13]. Although with some limitations, it has been demonstrated to assist in outbreak investigation [14] as shown by Bar-Meir et al. [15] in a neonatal intensive care unit. Additionally, MALDI-TOF MS has been used to identify antimicrobial resistance markers [16]. However, it is currently limited to identifying the isolates species and does not give details on the sequence type, serotype, and MLST clonal complex (CC). Although very useful, such an approach would not easily link isolates in an outbreak situation. The gold standard for this is whole-genome sequencing (WGS; Table 1) [7]. WGS allows high-throughput analysis of entire bacterial genomes, enabling extraction of information on phylogenetic relatedness, antibiotic resistance, virulence traits, serotype, and MLST of an isolate from a single analysis. On the food processor’s side, WGS allows efficient tracking of pathogen entry and distribution routes enabling adjustments to limit entry and spread [17,18]. However, it is restricted in terms of cost and time, taking an average of 2 to 4 days from colony identification to results. Additionally, skilled researchers with bioinformatic knowledge are required to analyze the genomes fully and to link them in an outbreak situation.

Fast and accurate pathogen detection is essential for correct disease diagnosis, treatment of infection, and reporting of infectious disease outbreaks and events, which is critical for controlling the course of an outbreak and avoiding large-scale epidemics and preventable loss of life [7,19,20]. The recently introduced IR Biotyper^®^ system (Bruker Daltonics GmbH, Bremen, Germany), a Fourier-Transform Infrared (FTIR) spectroscopy-based commercially available microbial typing system, is a good candidate for such low-cost, simple, and rapid routine use in outbreak investigation. Through FTIR spectroscopy, this system distinguishes strains by quantifying the absorption of infrared light by carbohydrates, lipids, nucleic acids, proteins, and lipopolysaccharides from microbial cells producing highly specific metabolic fingerprint-like signatures [8,21]. Several studies have shown that FTIR spectroscopy has high discriminatory power, enabling differentiation at the species or subspecies level [10,22,23]. It has been successfully employed for strain typing of pathogens such as *Legionella pneumophila*, *Pseudomonas aeruginosa*, *Streptococcus pneumoniae*, *Bacillus cereus* group, and *Staphylococcus aureus* [21,24,25,26,27]. A variety of FTIR-based methodologies have been utilized across microbial studies for tasks beyond microbial identification, such as process monitoring, cell wall analysis, biofilm examination, stress response assessment, and investigation of environmental interactions (reviewed by [23,28]). This versatility underscores the wide-ranging applications of FTIR spectroscopy in understanding microbial systems.

In FTIR spectroscopy, various instruments and methodological approaches have been employed when analyzing similar biological samples [28]. This paper highlights the potential of the IR Biotyper^®^ as a strain characterization tool that can be applied in place of traditional typing methods. Moreover, it covers sample preparation, data acquisition, and analysis techniques to ensure reliable and reproducible results.

## 2. Principle of FTIR Spectroscopy

**Figure 1:**
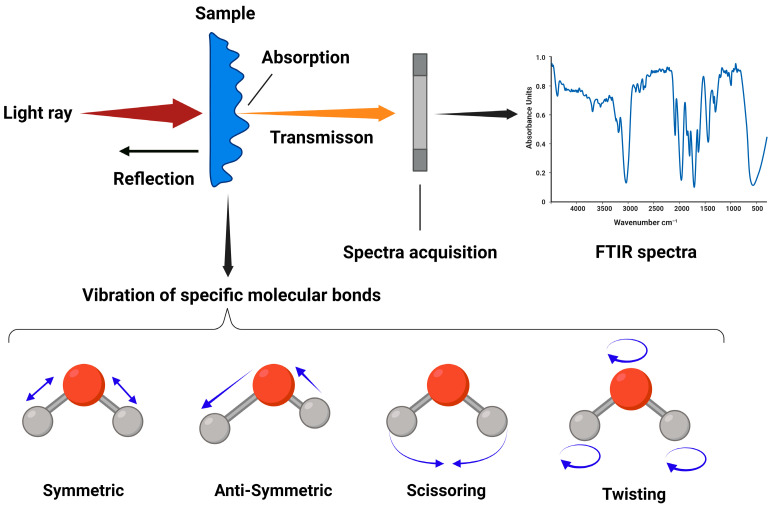
Infrared spectroscopy measures the interaction of infrared radiation with matter. Upon interaction with infrared light, molecular bonds within a sample vibrate (symmetric, antisymmetric, scissoring, rocking, wagging, or twisting vibration), absorbing specific wavelengths of light. The transmitted light carries chemical information of the sample, detected to produce an infrared spectrum with peaks corresponding to different chemical bonds. This infrared spectrum offers insights into the sample’s functional groups, enabling qualitative and quantitative analysis of its chemical composition, facilitating bacterial or yeast strain typing. Figure created with BioRender.com.

Infrared spectroscopy is based on the principle of measuring the absorption of infrared radiation by molecules [8]. Each molecule absorbs specific frequencies of infrared light that match the vibrational frequencies of their chemical bonds [23]. When infrared light interacts with a sample (bacterial or yeast cells), these wavelengths are absorbed by the sample’s molecular bonds, causing them to vibrate (Figure 1). The resulting spectrum, known as an infrared spectrum, contains peaks corresponding to the vibrational modes of different chemical bonds present in the sample.

In FTIR spectroscopy, infrared light is passed through the sample, and the transmitted light is collected and analyzed using an interferometer [10]. The interferometer produces an interferogram, which is then Fourier-transformed to generate the sample’s infrared spectrum. This spectrum provides information about the functional groups present in the sample, enabling qualitative and quantitative analysis of its chemical composition. For bacteria or yeast strain typing, FTIR spectroscopy can characterize the biochemical composition of cells, serving as their molecular fingerprints. Each strain exhibits a unique FTIR spectrum due to variations in its cell wall composition, membrane structure, and intracellular components. By comparing the FTIR spectra of different isolates, strains can be differentiated and classified according to their spectral signatures.

## 3. Protocol for IR Biotyper^®^-Based FTIR Spectroscopy Analysis

To benefit from the selectivity of FTIR spectroscopy and ensure reliable reproducible results, it is critical to adhere to a standard procedure for sample preparation using appropriate growth media at defined incubation temperature and duration.

### 3.1. Culture Preparation

To begin, strains must be resuscitated from cryo stocks by plating them on appropriate media and incubating them under specified conditions. For instance, *L. monocytogenes* can be plated on brain heart infusion (BHI) agar and incubated aerobically for 24 h at 37 °C. For FTIR analysis, starting with resuscitated cultures of the same age, pure single colonies are selected for subculture on appropriate media and incubated for specific time and temperature (Table 2). Strains should be streaked onto agar plates to achieve confluent growth. Depending on the species, additional subculture and incubation may be required to adapt the organisms to the growth conditions and increase cell mass. For liquid cultures, single colonies from each strain should be inoculated into 10 mL of broth and incubated under specific conditions. For example, *L. monocytogenes* can be grown in BHI broth and incubated for 24 h at 37 °C with shaking at 150 rpm.

The choice of media, temperature, and incubation time must be standardized to leverage the high selectivity of FTIR spectroscopy effectively. While blood agar and chromogenic agar have been used successfully, their use is generally discouraged. Blood agar can introduce additional variance, reducing discriminatory power, and chromogenic agar can colorize the biomass, altering infrared absorbance characteristics. Additionally, old or dried-out agar plates should be avoided, as variations in salt and nutrient concentrations can impact the growth and phenotypes of the target organisms.

### 3.2. Sample Preparation and Assay Setup

**Figure 2:**
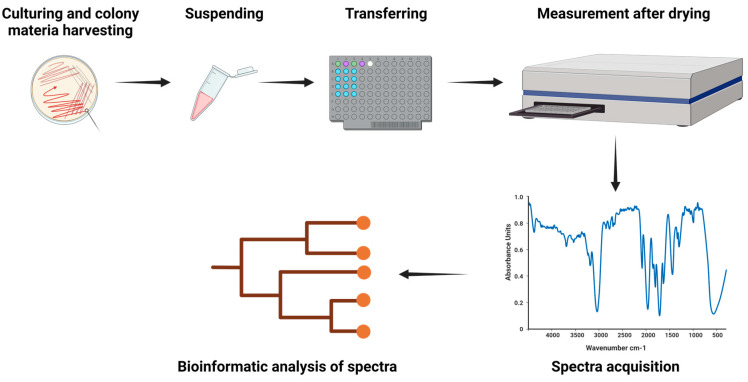
Sample processing protocol. A full loop of microbial colony material is collected from overnight cultures and resuspended in 70% ethanol in suspension vials. Care is taken to avoid agar transfer. After vortexing and addition of molecular grade water, 15 μL of the suspension is spotted in quadruplicate onto a 96-well silicon target plate and allowed to dry. Quality controls (IRTS 1 and IRTS 2) are prepared similarly. The dried plate is then inserted into the IR Biotyper^®^ for FTIR spectroscopy analysis. Post-measurement, spectra undergo quality assessment, and those meeting criteria are analyzed using dedicated software. Figure created with BioRender.com.

For spectra acquisition using the IR Biotyper^®^ (Bruker Daltonics GmbH & Co. KG), the manufacturer’s instructions for sample preparation and spectra acquisition are recommended (Figure 2).

For samples grown on agar, a full 1 μL loop of microbial colony material is collected from the confluent part of the plate culture and resuspended in 50 μL of 70% ethanol solution in a 1.5 mL suspension vial (IR Biotyper^®^ kit, Bruker Daltonics). It is important not to transfer any agar into the sample. After sufficient vortexing, 50 μL of molecular grade water will be added.

For broth cultures, it is essential to ensure that the pellet is thoroughly washed to remove any residual media. Depending on the species and growth level, 5–10 mL of culture should be centrifuged at 6000× *g* for 5 min. After discarding the supernatant, the cells should be washed twice with 5 mL of sterile phosphate-buffered saline (PBS), each followed by 5 min of centrifugation at 6000× *g*. Finally, the washed cells should be resuspended in 500 μL of PBS buffer for IR Biotyper^®^ measurement. The amount of culture harvested and the amount of PBS used for final resuspension can be adjusted depending on the absorbance values, which must fall in the range of 0.4–2.

For both samples from agar and broth, 15 μL of the suspension will be spotted in quadruplicate onto the 96-well silicon IR Biotyper^®^ target plate (Bruker Daltonics, Germany) and incubated at room temperature until the spots are dry (approximately 30–40 min). Because water molecules strongly absorb infrared light and can distort the spectra [28], sample spots must be sufficiently dried. However, care must be taken not to over-dry the sample spots, as this can cause cracks, leading to increased background noise and poor-quality spectra. The quality controls, Infrared Test Standards (IRTS 1 and IRTS 2) of the IR Biotyper^®^ kit, are resuspended in 100 μL of molecular grade water and mixed at 1500 rpm for 15 min at room temperature, then 60 μL of absolute ethanol is added with further mixing. Ten μL of the suspension are then spotted in duplicate onto the IR Biotyper^®^ target and left to dry as described for the samples. One well is left unused to act as the blank or background reading reference. Thereafter, they will be subjected to infrared spectroscopy analysis. All spectra are acquired intercalating a background spectrum between each sample/control measurement. Two to three independent biological replicates are best for each setup. From our experience, some microbes cannot be easily resuspended in ethanol; hence, they can be resuspended in water alone. Alternatively, the isolates can be resuspended in water first then sterilized with an equal amount of 70% ethanol.

### 3.3. Spectra Acquisition and Data Analysis

Using the IR Biotyper^®^ instrument, absorption spectra are recorded in transmission mode between wave numbers 4000 and 500 cm^−1^ using the OPUS software (Bruker Optics, Bremen, Germany). The sample plate containing the dried suspension spots is inserted into the instrument’s measurement chamber, which is continuously purged with dried air. It is crucial that the room housing the instrument maintains a stable climate, that is, humidity and temperature. The manufacturer’s acquisition method performs 32 scans for background and sample spectrum taking approximately 1 min per well. A maximum of 30 samples, each with 3 technical replicates, can be analyzed per silicon plate. Results can be obtained within 2–3 h from the time of culture harvest.

**Figure 3:**
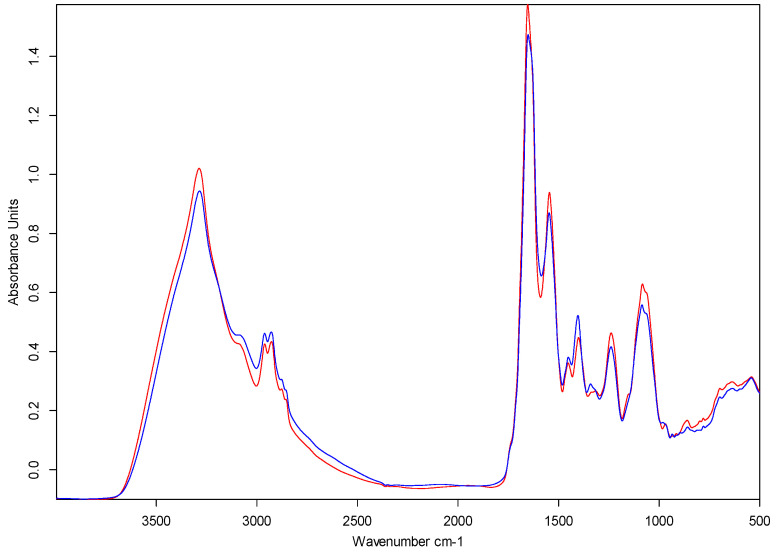
For visualization purposes, spectra of representative *L. monocytogenes* serotype 1/2a (blue) and 4b strains (red) produced using the OPUS software (Bruker Optics, Germany) are presented. Strains were cultivated on sheep blood agar at 37 °C for 24 h.

**Figure 4:**
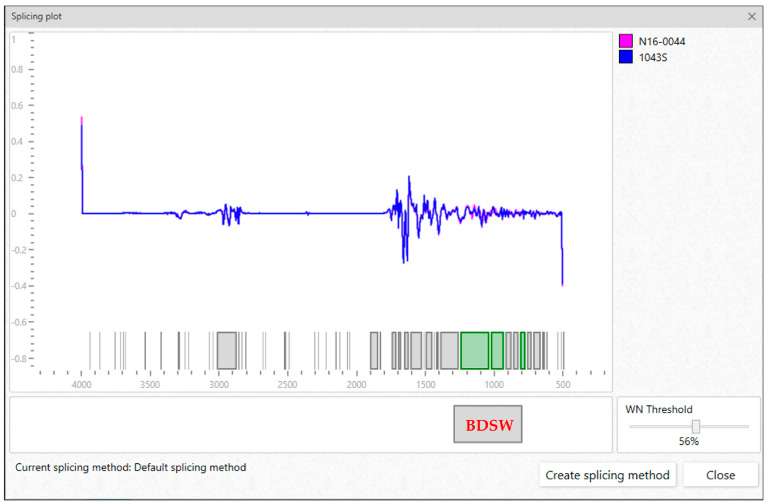
For visualization purposes, a second derivative spectra plot of representative *L. monocytogenes* serotype 1/2a (1043S) and 4b (N16-0044) strains, as seen in the IR Biotyper^®^ Client interface software, are presented. The strains were cultivated on sheep blood agar at 37 °C for 24 h. The grey rectangle labeled BDSW marks the IR Biotyper^®^ default splicing window: 1300–800 cm^−1^.

After measurement, the resulting spectra undergo a quality test. Spectra of poor quality, such as those with inadequate minimum and maximum absorbance values (outside the range of 0.4–2) and signal-to-noise ratio (R2 < 200 and R3 < 40), are identified. It is recommended to remove these spectra, allowing only those of acceptable quality for further analysis. A user-friendly IR Biotyper^®^ Client interface software (Bruker Daltonics) facilitates spectrum processing. The spectra (Figure 3) are smoothed using the Savitzky–Golay algorithm and the second derivative of the spectra is calculated. The spectra are then cut to the relevant spectral window/s and vector-normalized to regulate preparation-related variance in biomass and, consequently, absorption. The current default relevant spectral window of 1300–800 cm^−1^ is recommended, which primarily covers carbohydrates. However, other splicing methods focusing on regions corresponding to lipids, proteins, or proteins and carbohydrates can be applied (Table 3). For a detailed description of other spectral windows, readers are referred to the review by Kassem et al. [23]. It is also possible to create splicing methods that cover areas with high variance in the second derivative, either manually or through the software automatically (see Figure 4).

All qualitatively acceptable spectra can then be classified using commercially available classifiers, or users can develop their own classifier and integrate it into the IR Biotyper^®^. For species with a classifier, such as *L. monocytogenes* [29], *Salmonella* spp. [30], *S. pneumoniae* [31], and *L. pneumophila* [26], the serogroup is also reported per well as the test is running, with a traffic light system indicating classification confidence (green: high, yellow: moderate, and red: low confidence).

**Figure 5:**
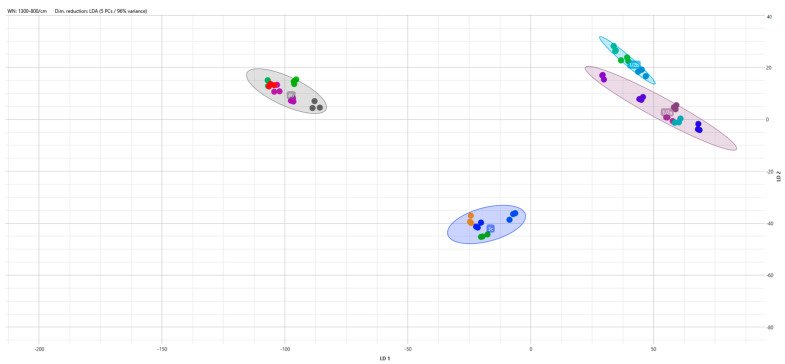
Two-dimensional scatter plot of Linear Discriminant Analysis (LDA) model using the serotype as a group identifier for *L. monocytogenes* strains. Spectra are color-coded by isolate, with each • representing an absorption spectra technical replicate. Wave number region 1300–800 cm^−1^ (polysaccharides).

The data can be analyzed using the Client interface software, employing multivariate statistical methods such as Hierarchical Cluster Analysis (HCA), Principal Component Analysis (PCA), and Linear Discriminant Analysis (LDA), allowing both supervised and unsupervised classification. Available linkage types include single, average, complete, and Ward’s linkage types, while clustering methods such as Euclidean and Pearson’s correlation are available. The data can be visualized as a dendrogram, distance matrix, and 2-D and 3-D scatter plots (Figure 5).

### 3.4. Key Considerations for FTIR-Based Protocols

To ensure accurate measurements, the FTIR spectrometer must be calibrated every 7 days using a Calibrator plate (Bruker Daltonics, Germany). The FTIR spectroscopy protocol might need to be optimized for each species. For instance, although this approach resulted in high discriminatory power similar to the ones obtained by *spa* typing and PFGE of *S. aureus* [32], the use of the IR Biotyper^®^ has been reported to be challenging for *S. aureus* strain typing. Optimization of the FTIR spectroscopy protocol, including reducing bacterial amount (from 1 µL to 0.5 µL loopful of bacterial culture), bacterial concentration (from 15 µL to 12 µL spotting), sample preparation, and appropriate media choice, was required to improve concordance of the results with WGS or PFGE data [33]. Wenning et al. [34] demonstrated that changes to the sample preparation procedure result in significantly impaired performance of FTIR spectroscopy, whereas they have fewer profound effects on MALDI-TOF MS. These observations indicate that FTIR spectroscopy might be strongly influenced by the sample preparation procedure, supporting the need for development of optimized species-specific protocols.

### 3.5. Limitations of FTIR Spectroscopy

FTIR spectroscopy, like other strain typing methods, is not immune to limitations [23,28]. For instance, sample preparation requirements, including the need for specific sample forms and processing, if not done properly, can introduce artifacts [35]. Its sensitivity to water, which strongly absorbs infrared radiation, poses constraints, particularly in analyzing water-rich samples. While FTIR can provide qualitative information about the chemical composition of a sample, achieving accurate quantitative analysis can be challenging. Factors such as sample thickness, homogeneity, instrumental parameters, and data analysis can affect the accuracy of these quantitative measurements.

The discriminatory power and concordance between FTIR spectroscopy results and WGS are not always consistently high. For instance, in the study by Zendri et al. [36], while the concordance between FTIR spectroscopy and MLST types was notably high for *K. pneumoniae* (Adjusted Rand Index [ARI] of 0.958), it was less satisfactory for *P. aeruginosa* (ARI of 0.313). This suggests that additional efforts and protocol refinement are required for certain species to enhance the performance of FTIR spectroscopy in strain typing. Hu et al. [37] demonstrated that the growth of *P. aeruginosa* isolates on Mueller–Hinton agar yields better discriminatory power compared to those grown on 5% sheep blood agar used in the study by Zendri et al. [36]. Additionally, while the equipment is affordable, there is a significant initial investment cost. However, with the high throughput of the machine, investment recovery can potentially be achieved within a short duration, especially for high throughput laboratories. Despite these limitations, FTIR spectroscopy remains valuable across disciplines, but researchers must be mindful of its constraints and employ complementary techniques where necessary.

## 4. Application of FTIR Spectroscopy

FTIR spectroscopy holds promise for various applications across different fields [23], including epidemiological investigations, contamination source tracking, and quality control of probiotics, starter, and ripening cultures [22]. However, it is crucial that regardless of the application, the results obtained through FTIR spectroscopy are consistent with WGS for phylogenetic clustering [38,39].

### 4.1. Outbreak Investigation

During outbreaks of infectious diseases, such as foodborne illnesses or healthcare-associated infections, identifying the specific strain responsible is crucial for implementing targeted control measures. Different strains of bacteria may exhibit varying transmission dynamics, virulence, and antibiotic resistance patterns. By employing strain typing techniques like FTIR spectroscopy, researchers and public health officials can quickly identify the causative strain, trace its source, and implement preventive measures to contain the outbreak. The IR Biotyper^®^ has demonstrated comparability to WGS in differentiating extended-spectrum β-lactamase-producing *Escherichia coli* and *Klebsiella pneumoniae* isolates [20,21,37,40,41]. In a prospective vancomycin-resistant *Enterococcus faecium* outbreak investigation, FTIR spectroscopy showed better concordance with WGS-average nucleotide identity (ANI) than MLST [42]. It has also shown applicability for real-time tracking and monitoring of multidrug-resistant *Acinetobacter baumannii* isolates from an intensive care unit outbreak [43].

### 4.2. Factory Hygiene Monitoring Programs

In industries where microbial contamination poses a risk to product quality and consumer safety, such as food processing and packaging, maintaining stringent hygiene protocols is essential. Pathogen strain typing enables the monitoring of microbial populations within manufacturing facilities, identifying potential sources of contamination, and implementing corrective actions to prevent product spoilage or contamination-related recalls. Rapid and reliable strain identification methods like the IR Biotyper^®^-based FTIR spectroscopy support real-time monitoring and quality assurance in factory environments, allowing for easy identification of persistent strains or pathogen reintroduction.

### 4.3. Food Production Quality Control

The IR Biotyper^®^ is also applicable for food quality control to verify if the starter culture strains added during production remain consistent throughout the process. It has been employed to accurately differentiate four *Lactiplantibacillus plantarum* probiotic strains [44]. In this study, it demonstrated high discriminatory power even when the strains were cultivated under different conditions. Interestingly, the growth medium (broth and agar) did not affect its ability to distinguish the four probiotic strains [44]. In fact, broth cultures exhibited higher reproducibility and discriminatory power compared to agar cultures. Analysis of *L. plantarum* strains demonstrated that FTIR spectroscopy was not only comparable to WGS and PFGE but also exhibited a higher discriminatory power than MLST [45]. Furthermore, Deidda et al. [46] showed that FTIR spectroscopy analysis is comparable to WGS, MLST, and PFGE in discriminatory power for *Bifidobacterium longum* subsp. *longum* strains, and superior to MLST and PFGE in differentiating *B. animalis* subsp. *lactis* strains.

### 4.4. Clinical Settings

The IR Biotyper^®^ provides rapid, cost-effective, and high-throughput strain typing, making it a valuable tool in clinical settings. Timely and accurate identification of pathogens is essential for diagnosing infectious diseases, connecting nosocomial pathogens to environmental sources, and initiating appropriate treatment and corrective measures [40]. Different bacterial strains may display variations in antibiotic susceptibility, virulence, and disease progression. Therefore, precise strain typing methods are essential for guiding therapeutic decisions, particularly in cases of antibiotic-resistant infections or outbreaks of multidrug-resistant bacteria. By quickly identifying the causative strain, the IR Biotyper^®^ can assist clinicians in tailoring treatment regimens to maximize efficacy and minimize the risk of treatment failure. For instance, Potocki et al. [47] demonstrated that FTIR spectroscopy holds promise for facilitating diagnosis and targeted therapy for candidiasis. FTIR spectroscopy has also shown significant discriminatory capability in veterinary hospital epidemiological surveillance of *K. pneumoniae* [36], thus enabling the timely deployment of effective infection control strategies in such settings. It has also been successfully applied to characterize Gram-negative bacilli (*A. baumannii*, *Enterobacter cloacae*, and *P. aeruginosa*) clones responsible for nosocomial outbreaks [25].

### 4.5. FTIR Spectroscopy in Basic Research

FTIR spectroscopy is applicable in basic research, particularly in the investigation of cellular responses to stress and genetic modifications [23]. By comparing the intensity of absorption peaks corresponding to different cellular components, researchers can quantify the amount of each component present in different strains. For instance, using high throughput FTIR spectroscopy, Smirnova et al. [48] demonstrated that altering temperature and nutrient levels impacts the metabolic processes and cellular chemical composition of bacteria isolated from Antarctic green snow.

## 5. Research Outlooks

Our current research is focused on utilizing the IR Biotyper^®^ for outbreak investigation and improving the current classifiers to enhance discriminatory power, particularly among closely related serogroups. A priority is achieving clear separation among *L. monocytogenes* 1/2a, 1/2b, and 1/2c serotype strains. Initial findings for *L. monocytogenes* show promising clustering and outbreak detection capabilities, especially when outgroup strains are distantly related to the outbreak cluster. However, we have observed a low discriminatory power (ARI of <0.4) when analyzing very closely related strains, indicating a potential limit of discrimination based on single nucleotide polymorphism differences.

Pathogens do not respect borders, highlighting the necessity for efficient disease surveillance at the local, national, and international levels [9]. One advantage of WGS is its ability to facilitate the creation of global databases based on standardized nomenclatures, such as the MLST databases. The benefit of such global databases lies in the international exchange of data, enabling cross-border outbreak investigation, strain tracking, and source identification in the global food chain [49,50].

It remains to be seen if a similar worldwide accessible database of FTIR reference spectra can be established for the IR Biotyper^®^, which would allow this tool to be applicable for international source tracking and multinational outbreak investigation. However, any efforts to create such a database must be accompanied by a standardized protocol for generating, storing, sharing, and analyzing FTIR spectra profiles to ensure comparability between different laboratories. For these databases to be effective, they would need to include a wide range of reliably identified reference strains to cover the intraspecies diversity of microbes [22]. A translational research approach is therefore needed to address these limitations through interdisciplinary collaboration and open data sharing. Additionally, further enhancement of the discriminatory power of the IR Biotyper^®^ is necessary, and the classifier database should be expanded to include other pathogens beyond the four currently available. Overall, further validation of this procedure across different pathogen species and by various laboratories is still required to facilitate widespread adoption.

## 6. Conclusions

This methodology provides a comprehensive guide for utilizing FTIR spectroscopy in bacteria and yeast strain typing. By following the outlined steps for sample preparation, FTIR data acquisition, and advanced data analysis techniques, reliable and reproducible results can be obtained, rendering this approach valuable across various microbiological applications.

In summary, the IR Biotyper^®^ offers a rapid, high-throughput, non-destructive (does not damage or alter the sample), environmentally friendly, cost-effective, and minimal hands-on-time tool for microbial strain typing, making it well suited for diverse applications in food microbiology, epidemiology, and clinical diagnostics. Through the utilization of FTIR spectroscopy, researchers and public health officers can advance the understanding of microbial diversity, enhance outbreak surveillance efforts, and improve strategies for disease prevention and control. This method might be applied as an early isolate screening or warning system, complemented by subsequent detailed analysis techniques such as WGS. Its adoption for microbial strain typing has the potential to revolutionize rapid-response scenarios in infection and outbreak management, as well as in food quality control and hygiene standards.

## Figures and Tables

**Table 1:** Comparison of strain typing methods.

Strain Typing Method	Discriminatory Power	Cost	Time
WGS	Highest	High	Lengthy
PFGE	High	Moderate to high	Lengthy
MLST	High ^1^	High	Moderate
Sera Serotyping	Moderate to high	Low to moderate	Moderate
FTIR	High ^2^	Moderate	Rapid

^1^ Limited to applicable strains. ^2^ Variable depending on species and sample preparation.

**Table 2:** Strain culture conditions.

Organism ^1^	Media	Temperature	Time	Atmosphere
*L. monocytogenes*	BHI, TSA ^**2a**^	37 °C	24 ± 0.5 h	Aerobic
*S. pneumonia*	Blood agar ^**3**^	37 °C	24 ± 0.5 h	Microaerophilic, capnophilic
*S. enterica*	TSA ^**b**^	37 °C	24 ± 0.5 h	Aerobic
*L. pneumophila*	BCYE ^**4**^	37 °C	48 ± 1 h	Microaerophilic, humid

^1^ Only species with a validated IR Biotyper^®^ classifier have been included. ^2^ TSA: Tryptic soy agar. ^3^ Contains 5% sheep blood. ^4^ BCYE: Buffered charcoal yeast extract agar. ^a^ Other media such as Sheep blood agar, ALOA, Oxford, Palcam, Rapid’ L. mono agar have been validated by the manufacturer. ^b^ Other media such as Sheep blood agar, Chocolate, Mueller–Hinton, XLD, Salmonella Shigella, MacConkey agar have been validated by the manufacturer.

**Table 3:** Spectral windows and their recommended applications.

Spectral Window	Recommended Application
1300–800 cm^−1^	Carbohydrates
3000–2800 cm^−1^	Lipids
1800–1500 cm^−1^	Proteins
1800–900 cm^−1^	Proteins and carbohydrates

## Data Availability

No new data were created or analyzed in this study. Data sharing is not applicable to this article.
